# Classification of Tandem Mass Spectra for Identification of N- and O-linked Glycopeptides

**DOI:** 10.1038/srep37189

**Published:** 2016-11-21

**Authors:** Shadi Toghi Eshghi, Weiming Yang, Yingwei Hu, Punit Shah, Shisheng Sun, Xingde Li, Hui Zhang

**Affiliations:** 1 Department of Biomedical Engineering, Johns Hopkins University, Baltimore, MD 21205, USA; 2 Department of Pathology, Johns Hopkins University, Baltimore, MD 21231, USA.

## Abstract

Analysis of intact glycopeptides by mass spectrometry is essential to determining the microheterogeneity of protein glycosylation. Higher-energy collisional dissociation (HCD) fragmentation of glycopeptides generates mono- or disaccharide ions called oxonium ions that carry information about the structure of the fragmented glycans. Here, we investigated the link between glycan structures and the intensity of oxonium ions in the spectra of glycopeptides and utilized this information to improve the identification of glycopeptides in biological samples. Tandem spectra of glycopeptides from fetuin, glycophorin A, ovalbumin and gp120 tryptic digests were used to build a spectral database of N- and O-linked glycopeptides. Logistic regression was applied to this database to develop model to distinguish between the spectra of N- and O-linked glycopeptides. Remarkably, the developed model was found to reliably distinguish between the N- and O-linked glycopeptides using the spectral features of the oxonium ions using verification spectral set. Finally, the performance of the developed predictive model was evaluated in HILIC enriched glycopeptides extracted from human serum. The results showed that pre-classification of tandem spectra based on their glycosylation type improved the identification of N-linked glycopeptides. The developed model facilitates interpretation of tandem mass spectrometry data for assignment of glycopeptides.

The ability of cells to co- and post-translationally modify proteins has created tremendous diversity in the protein forms and functions. Glycosylation, which is the covalent attachment of sugar molecules to proteins, is one of the most common of these modifications and accounts for the greatest diversification of proteins. The glycosylation biosynthesis is utterly sophisticated, determined by numerous factors including expression of biosynthetic enzymes, presence of glycosylation sites on proteins, and availability of monosaccharide substrates^1^,^2^. Common types of protein glycosylation include N- and O-linked glycosylation. N-linked glycosylation (N-glycosylation) mainly occurs on the consensus motifs NXS/T and less commonly on NXC and NXV sequences^3^, where X can be any amino acid except proline. N-linked glycans (N-glycans) have a common core consisting of two N-acetylglucosamine (GlcNAc) and three mannose (Man) residues, and can be subdivided into high-mannose, complex and hybrid structures^4^. N-glycosylation is particularly common on secreted, membrane-bound or cell surface proteins^5^. O-linked glycosylation (O-glycosylation) mainly occurs on S/T or less commonly Y residues on proteins^6^. O-linked glycan (O-glycan) structures are more diverse than N-linked glycans and include subclasses of O-GalNAc, O-fucose and O-mannose, where for O-GalNAc – also known as mucin-type — glycans alone, 8 core structures have been described^4^. Mucin-type glycoproteins, which are found in excess in mucous, contain S/T rich regions that are heavily O-glycosylated. These regions usually are rich in proline as well, which subsequently facilitates the O-glycosylation at S/T residues^7^. Mucin-type glycoproteins are believed to provide immunity against pathogens by serving as decoys to their lectin receptors^8^. Another form of protein glycosylation on S/T, called O-GlcNAcylation, is the attachment of a GlcNAc to the S or T residues on proteins by the O-GlcNAc transferase (OGT) enzyme and can happen in cytoplasm, nucleus or mitochondria. O-GlcNAcylation is a dynamic modification in nature and can be reversed by detachment of the GlcNAc from the peptide using the O-GlcNAcase (OGA) enzyme, which makes the S or T residue available for other modifications such as phosphorylation^9^. Additionally, glycosaminoglycans (GAGs) are long polysaccharide chains composed of repeated disaccharide units that could also modify S/T residues on protein sequences creating proteoglycans^10^.

High-throughput investigation of protein glycosylation is regularly achieved through mass spectrometry analysis. Previously, these studies mainly involved enrichment or isolation of glycopeptides using hydrazide chemistry, lectin affinity techniques or hydrophilic interaction chromatography (HILIC), which result in identification of glycans and glycosite-containing peptides^11^,^12^,^13^,^14^,^15^. However, site-specific glycosylation microheterogeneity plays key roles in the function of this post-translational modification^16^,^17^,^18^,^19^ and therefore the ability to preserve this information is critical. Hitherto advances in mass spectrometry techniques has facilitated the detection of intact glycopeptides from recombinant proteins, glycoprotein cocktails or complex biological samples^18^,^20^,^21^,^22^,^23^,^24^,^25^,^26^. However, mass spectrometry data is being generated at a rate as high as one gigabytes/hour on a single instrument, resulting in massive amounts of glycoproteomics data being collected. The subsequent data analysis would be undermined if software tools and algorithms were unable to extract compelling but hidden information from the data.

Many efforts in the glycoproteomics field have focused on N-glycosylation^18^,^20^,^23^,^24^,^25^,^26^,^27^. Higher-energy collisional dissociation (HCD) fragments the glycopeptides both at glycosidic and peptide bonds, generating glycan oxonium ions, peptide fragment ions and peptide ions with partial glycans^26^. This fragmentation method, alone or in combination with others, has proven to be effective in fragmentation and identification of N-glycoproteins^18^,^26^,^28^,^29^,^30^. However, the use of HCD for analysis of O-glycoproteins remains largely unexplored. Oxonium ions, which are a result of fragmentation of mono- or disaccharides from the glycopeptides, contain structural information that could facilitate accurate assignment of glycopeptides. The abundance of different oxonium ions depends on the composition of glycans as well as their structure^31^. N- and O-glycosylation are different in their peptide-glycan linkage, and glycan composition and structure^1^. Due to these differences, oxonium ions from each of these classes of glycopeptides are likely to exhibit distinct patterns and characteristics.

Here, we studied the characteristic features of oxonium ions originated from different N- and O-linked glycopeptides. Logistic regression is used to determine the glycosylation type based on the intensities of 10 oxonium ions from fragmentation of hexose, N-acetylhexosamine, and sialic acid residues from glycans. It was found that oxonium ions in the MS/MS spectra of glycopeptides could be used to efficiently distinguish N- and O-linked glycopeptides in glycoprotein standards and human serum samples. Examining oxonium ions for revealing structural details of glycopeptides facilitates confident assignment of glycopeptides to tandem spectra and reduces the size of search space, thus improving the performance of the data mining process. This classification tactic has been incorporated into GPQuest^26^. Together with a refined decoy and scoring algorithm, GPQuest has been further improved to identify site-specific N- and O-glycosylation in the HCD MS/MS spectra of intact glycopeptides.

## Methods

### Sample preparation and mass spectrometry analysis

#### Materials and reagents

Sequencing-grade trypsin was purchased from Promega (Madison, WI). ZIC-HILIC columns were from The Nest Group (Southborough, MA). C18 desalting cartridges were purchased from Waters (Milford, MA). Glycophorin A (P02724), bovine fetuin (Q58D62 and P12763), ovalbumin (P01012) and human serum were purchased from Sigma Aldrich. All other reagents were purchased from Sigma Aldrich (St. Louis, MO) unless otherwise specified.

#### Glycopeptide sample preparation

The glycoproteins of interest, glycophorin A, ovalbumin, and fetuin and human serum were first denatured using 8 M urea in 1 M ammonium bicarbonate buffer with 10 mM tris(2-carboxyethyl)phosphine (TCEP) for one hour at room temperature. Denaturing was followed by alkylation using iodoacetamide for 30 mins at room temperature in the dark. The alkylated glycoproteins were then digested using high-grade trypsin and incubated overnight at 37 °C. The peptides were purified using C18 columns. The peptide samples were prepared and analyzed in duplicates. The first run was used to build the training dataset and the model to classify N- and O-glycosylation and the second run was used as a testing dataset to verify the application of the prediction model. For human serum, N- and O-glycopeptides were enriched by HILIC columns from tryptic peptides that were prepared as described above. For HILIC enrichment, the cartridges were conditioned three times with solutions of 1% TFA and 80% ACN/1% TFA. Peptides in 80% ACN/1% TFA were loaded to the cartridges and washed three times with 80% ACN/1% TFA. Glycopeptides were eluted in 400 μl of 0.1% TFA.

### Mass spectrometry analysis

For each glycoprotein, a 0.4 *μ*g aliquot and for the human serum sample a 1 μg aliquot was separated through a C18 column on a Dionex Ultimate 3000 RSLC system and analysed on a Q Exactive mass spectrometer (Thermo Scientific). Data-dependent HCD fragmentation was performed on the 10 most abundant ions. The spray voltage was set to 1.8 kV. Spectra (AGC target 1 × 10^6^ and maximum IT 60 ms) were collected from 400–2000 m/z at a resolution of 70 K followed by data-dependent HCD MS/MS (at a resolution of 17,500, NCE 29, intensity threshold of 5 × 10^4^, AGC target 1 × 10^5^ and maximum IT 100 ms) of the 10 most abundant ions using an isolation window of 2 *m*/*z*. Charge state screening was enabled to reject unassigned, singly and more than eight protonated ions. A dynamic exclusion time of 10 sec was used to discriminate against previously selected ions.

### Data analysis

#### N- and O-glycopeptide MS/MS spectra dataset

The mass spectrometry raw files of fetuin, ovalbumin and glycophorin A were converted to mzXML files using the Trans-Proteomic Pipeline (TPP)^32^, with the “centroid all scans” option selected. The precursor mass matching algorithm on GPQuest was used to analyse the mzXML files. The following parameters were used in GPQuest: Mass tolerance was set to 10 and 20 ppm at MS1 and MS2 levels, respectively. N- and O-glycosylation analysis were conducted simultaneously. In house N- and O-glycan databases each contained 253 and 143 compositions, respectively. *In silico* tryptic digestion of the amino acid sequences of these three glycoproteins with UniProt IDs of Q58D62, P12763 (fetuin), P02724 (glycophorin A) and P01012 (ovalbumin) was used to generate the peptide database for this analysis. Up to two missed cleavages and maximum two variable oxidation of Methionine were allowed. An MS/MS spectrum was marked as oxonium-ion containing for further glycoproteomics analysis if it contained a minimum of 2 oxonium ions in the 5% highest peaks, with the ion at 204 being mandatory as one of the two. The bottom 10% of the peaks were removed as low-quality peaks. Singly charged b and y peptide fragment ions were included for scoring of the glycopeptide-spectral matches (GPSM). For peptides shorter than 11 amino acids, presence of the intact peptide ion was mandatory. The false discovery rate (FDR) was set to 1%. To estimate the false discovery rate, a decoy strategy was used. In this strategy, the decoy database was built by concatenating and shuffling the amino acids of the target database and dividing the generated sequence into decoy peptides with lengths similar to the target peptides^26^. Several additional filters were applied to GPQuest output to ensure that the spectra selected for the training dataset were of the highest quality with confident glycopeptide assignments. First of all, only GPSMs including peptides with known glycosylation sites were selected. Also, GPSMs with intensity coverage below 10% and total spectrum intensity below 5 × 10^5^ were excluded from the dataset, where total spectrum intensity was the sum of all peak intensities in the MS/MS spectrum. For spectra of O-glycopeptides, only GPSMs assigned to peptides lacking any N-glycosylation sites were considered.

#### Scoring of glycopeptide-spectral matches

A modified version of the Morpheus score reported by Wenger *et al*. for peptide-spectral matches in high mass resolution proteomics analysis^33^ was used in scoring of GPSMs using GPQuest. The original Morpheus score reports the sum of the number of fragment b and y ions and the intensity fraction of the tandem MS spectrum covered by the matching fragment ions. For glycoproteomics analysis, we have modified the score in two ways. First, in addition to the fragment b and y ions, this score includes the number of intact peptide ions such as peptide^+^ or peptide-HexNAc^+^, which can be major peaks in HCD MS/MS spectra of glycopeptides. Second, the fractional part of the score is the intensity fraction of b, y and intact peptide ions with or without partial glycans after removing the oxonium ions from the spectra. Excluding the oxonium ion intensities ensures that these ions, which are disproportionately abundant in the spectra of N-glycopeptides, do not interfere with the scoring of the GPSMs.

#### Data analysis of human serum samples

GPQuest was used to analyse the input mzXML file of the serum glycopeptides. The N-glycan database was curated by combining databases from Functional Glycomics Gateway (CFG)^34^, GlycoWorkbench^35^, and GlycomeDB^36^ and the O-glycan database was obtained from CFG^34^. A human serum-specific N-linked peptide database that was generated using solid phase extraction of glycosite-containing peptides (SPEG)^11^,^37^ and solid phase extraction of N-linked glycans and glycosite-containing peptides (NGAG)^18^ in previous studies was used. The O-linked peptide database was also from previous studies^38^,^39^. A minimum of two oxonium ions in top 10% of peaks in the spectra was required in an MS/MS spectrum to qualify for glycopeptide search. No intact peptide ions or intact peptide ions with partial glycans were required. Other parameters were the same as the parameters used for analysis of the glycoproteins.

## Results and Discussion

### Glycosylation type classification of MS/MS spectra using oxonium ions

**Figure 1:**
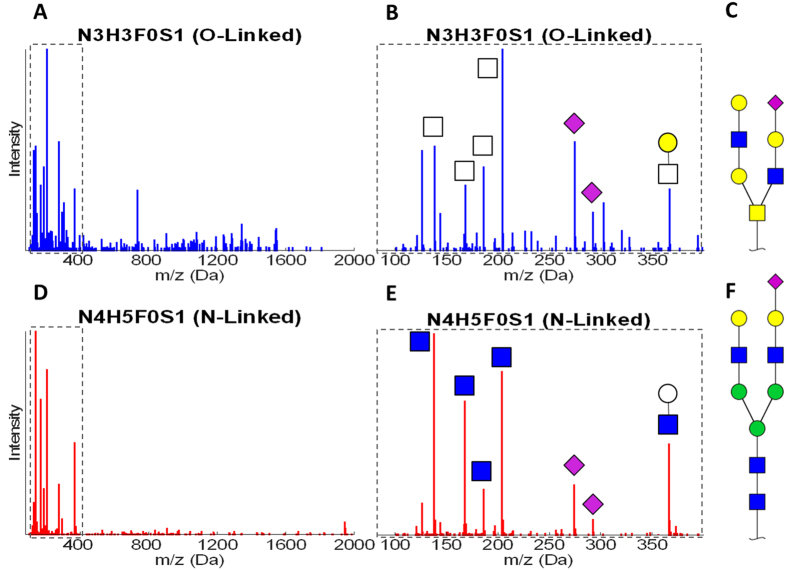
Intensity of glycan oxonium ions differs between HCD fragmented O- and N-glycopeptides. The MS/MS spectra of HCD fragmented glycopeptides with (**A**) O- and (**D**) N-glycans are shown. Focusing on the lower mass range that covers the oxonium ions for (**B**) O- and (**E**) N-glycans shows that these two glycosylation types create very different patterns of fragment ions in this range for the N-linked N3H3F0S1 (**C**) and O-linked N4H5F0S1 (**F**) glycan structures. The illustrated glycans depict possible structures for each glycan composition. There are several differences in the intensities of the oxonium ion between these two glycopeptides. Among these differences are the intensity ratios of HexNAc oxonium ions at 138, 168, 186 and 204, the intensities of Neu5Ac oxonium ions at 274 and 292 and the intensity of HexHexNAc oxonium ion at 366. Glycoworkbench^35^ was used for creating the glycan structure figures. Blue square: GlcNAc; yellow square: GalNAc; white square: HexNAc; yellow circle: Galatose; green circle: mannose; white circle: Hexose; purple diamond: N-Acetylneuraminic acid (Neu5Ac).

The MS/MS spectra of glycopeptides carry important structural information. In HCD mode, various types of glycopeptide fragment ions are produced. An important signature of glycopeptide spectra is the presence of oxonium ions that are mono- or disaccharides fragmented from the glycan moieties. These oxonium ions not only distinguish glycosylated from non-glycosylated peptides, but also shed light on the glycan structure^31^. We have observed that in particular, the oxonium ions fragmented from N- or O-glycopeptides were quite different in their intensities and distribution. Figure 1 shows the MS/MS spectra of two glycosylated peptides. The top panel shows the spectrum of the peptide ‘VTCTLFQTQPVIPQPQPDGAEAEAPSAVPDAAGPTPSAAGPPVASVVVGPSVVAVPLPLHR’ which is a tryptic peptide from bovine fetuin and has known O-glycosylation sites. The spectrum shows that this peptide is modified by an O-glycan with composition of N3H3F0S1, where N, H, F and S represent the number of HexNAc, Hexose, Fucose and Neu5Ac residues, respectively. The bottom panel depicts the fetuin N-glycopeptide ‘LCPDCPLLAPLNDSR’ that has an N-glycosylation motif and has been modified by an N-glycan with composition N4H5F0S1. Both glycan compositions are sialylated and non-fucosylated. They consist of 7 and 10 monosaccharides respectively. The figure also shows the *m*/*z* range spanning the oxonium ions and their intensities. Seven oxonium ions are annotated on this figure including *m*/*z* at 138, 168, 186 and 204 (HexNAc ions), 274 and 292 (Neu5Ac ions) and 366 (HexHexNAc ions). There are a few noteworthy distinctions between the two spectra. First, the pattern of the HexNAc ion intensities is significantly different between spectra of N- and O-glycopeptides. Second, the two ions corresponding to Neu5Ac have a higher share of the total intensity in the O-linked spectrum compared to the N-linked spectrum, while the opposite is true for the HexHexNAc ions. These distinctions in oxonium ions were also observed in the spectra of other N- and O-linked glycopeptides.

To systematically study the differences between spectral features of N- and O-glycopeptides, a training dataset was generated that contained HCD fragmented spectra of different glycopeptides. This dataset was built by first analyzing the glycoproteomic data of ovalbumin, glycophorin A and fetuin using GPQuest and selecting confidently assigned GPSMs as described in the Methods section. Ovalbumin was included in this experiment because it is an N-glycoprotein, with no reported O-glycosylation sites and therefore provided N-glycopeptide spectra for the training dataset. Glycophorin A, in contrast, is heavily O-glycosylated and therefore provided many O-glycopeptide spectra. Ideally, the training dataset should be diversified to cover a wide variety of possible cases. The size and diversity balance of the O-linked and N-linked entries in the database were improved by adding 79 manually verified glycopeptide spectra from our study of gp120 glycosylation^30^ to this dataset. Concatenation of the data from ovalbumin, glycophorin A, fetuin and gp120 resulted in a training dataset containing 872 and 527 HCD fragmented spectra of N- and O-glycopeptides, respectively (supplementary Table S1).

**Figure 2:**
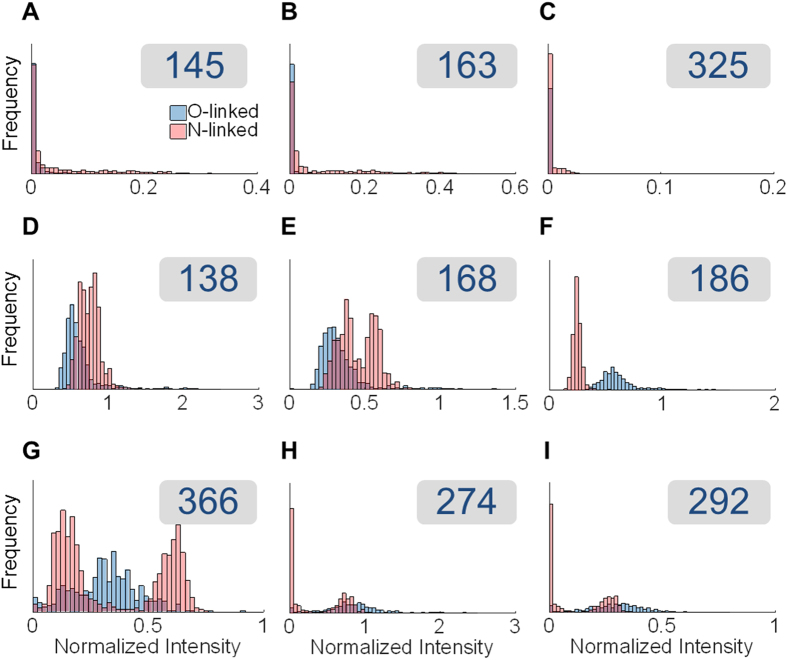
Spectral differences between O- and N-glycopeptides in the oxonium ion region. The intensities of 9 different oxonium ions normalized by the intensity of HexNAc ion at 204 are depicted. The plotted ions are as follows: (**A**) Hex—H_2_O ion at 145, (**B**) Hex ion at 163, (**C**) Hex_2_ ion at 325, (**D**) HexNAc internal fragment ion at 138, (**E**) HexNAc—2 H_2_O ion at 168, (**F**) HexNAc—H_2_O ion at 186, (**G**) HexHexNAc ion at 366, (**H**) Neu5Ac—H_2_O ion at 274 and (**I**) Neu5Ac ion at 292. There are clear differences between the two types of glycosylation in terms of their oxonium ion intensities. These differences are more pronounced for some of these ions. In particular, normalized intensity of 186 is significantly higher in O-linked spectra. The other two HexNAc ions are however higher in the N-linked spectra. Furthermore, sialic acid residues have a greater share in the oxonium ion intensities of O-linked spectra while Hex oxonium ions are generally higher in the N-linked spectra. These differences, fed into a machine learning algorithm, can help classify the spectra and predict the glycosylation type.

Figure 2 illustrates the intensities of oxonium ions in N- and O-glycopeptide MS/MS spectra side by side. The intensities were extracted for nine oxonium ions and normalized by the intensity of the HexNAc ion at m/z 204, which is observed in almost all the spectra that belong to glycopeptides. The distributions of the oxonium ion intensities are plotted for N-linked (in red) and O-linked (in blue) spectra. As shown, there are significant differences between the two types of glycosylation: 1) Hexose oxonium ions (at *m*/*z* 145, 163 and 325) are usually more abundant in the spectra of N-glycopeptides. 2) The oxonium ions for sialic acid (at *m*/*z* 274 and 292) are more abundant in the spectra of O-glycopeptides. 3). There is a clear difference in the normalized intensities of different HexNAc oxonium ions between the spectra of N- and O-glycopeptides. Particularly, the intensity of the ion at *m*/*z* 186 normalized by that of the ion at *m*/*z* 204 is significantly higher in the spectra of O-glycopeptides compared to those of N-glycopeptides.

Spectral features can be used to distinguish spectra of N- and O-glycopeptides using machine learning. We employed multinomial logistic regression classification^40^, and trained and cross-validated this classifier on our curated spectral dataset of N- and O-glycopeptides. The intensities of the 9 aforementioned oxonium ions in Fig. 2 normalized by the intensity of the ion at *m*/*z* 204 were chosen as the inputs to the classification algorithm. The use of the normalized intensities makes this approach more transferable to other platforms, as different manufacturers and instruments use different units of measurement for ion intensities. Classification on this dataset of 1399 spectra from gp120, glycophorin A, ovalbumin and fetuin, yielded an overall accuracy of 98.8%, where the true ‘N-linked rate’ was 99.7% and the true ‘O-linked rate’ was 97.3%. The classifier was additionally validated on a second dataset of spectra from the repeated analysis of fetuin, glycophorin A and ovalbumin with 94.4% accuracy, indicating the reproducibility of these spectral features. One of the advantages of using logistic regression is that in addition to classifying the spectrum, it provides the numerical probability of the classification being correct. This attribute is particularly crucial for the spectra that lie within the gray zone between the two types of glycosylation. Statistics of the model coefficients showed that the most crucial feature for classification was the intensity ratio of ions at *m*/*z* 186 to 204 with a p-value close to zero. Compared to N-glycans, O-glycans have proportionally more GalNAc residues, whereas N-glycans have relatively more GlcNAc residues. The differences in the ratio of these HexNAc ions suggest that GalNAc and GlcNAc may be fragmented dissimilarly in HCD mode. Halim *et al*. showed that the intensities of the oxonium ions in glycopeptide MS/MS spectra helped identify the glycan saccharide identities and in fact they demonstrated that the ratio of 186 to 204 oxonium ions is higher for GalNAc containing glycans compared to their GlcNAc containing counterparts^31^, which is in line with our observation.

Fragmentation differences between stereoisomers, such as GlcNAc and GalNAc, are one of the possible explanations for the distinct spectral patterns of oxonium ions in MS/MS spectra of N- and O-glycopeptides. Other factors may include difference in the size and branching pattern of glycans. In general, N-glycans are larger in size and tend to be more branched compared to O-linked ones. Figure 2G depicts the distribution of the intensity ratio of ions at *m*/*z* 366 to 204 in spectra of N- and O-glycopeptides in the training dataset. The average normalized intensity of the oxonium ions at *m*/*z* 366 for several high-mannose and complex N-glycans and O-glycans is depicted in supplementary Table S2. In the spectra of N-glycopeptides, the oxonium ions at *m*/*z* 366 represent HexHexNAc disaccharides. In N-glycans, these HexHexNAc ions could correspond to the ManGlcNAc duo in the core of any glycan or the ManGlcNAc or GalGlcNAc saccharide pairs on the branches of complex or hybrid glycan structures. According to Table S2, high-mannose N-glycans show relatively lower levels of HexHexNAc oxonium ions compared to complex N-glycans with higher numbers of terminal GalGlcNAc branches, whereas O-glycans with similar compositions lie between the aforementioned groups. Additionally, the number of terminal GalGlcNAc branches in N-glycans is positively correlated with the average intensity of the HexHexNAc ions (r = 0.72). We hypothesize that in branched complex N-glycans, the structurally parallel nature of these branches allows for the disaccharides to fragment independently and generate more intense peaks at *m*/*z* 366. In O-glycans, the oxonium ions at *m*/*z* 366 are likely to be coming from a limited number of HexHexNAc disaccharides available for fragmentation. Taken together, the spectral differences of oxonium ions between N- and O-glycopeptides could be a result of differences between fragmentation of stereoisomers, differential responses of N- and O-glycans to HCD fragmentation, as well as structural characteristics of the glycan branches.

**Figure 3:**
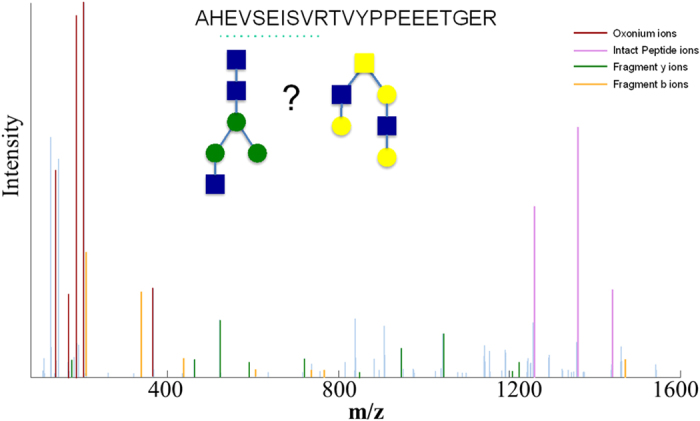
Prediction of the glycosylation type. The MS/MS spectrum corresponding to the following GPSM is shown: AHEVSEISVRTVYPPEEETGER – N3H3F0S0. The glycan composition of this GPSM can represent an N- or O-linked structure. The intensity of the oxonium ions, particularly the high ratio of 186 to 204 implies the glycosylation type to be O-linked. This prediction is in agreement with the peptide sequence, which lacks an N-glycosylation site and can only be O-glycosylated. The blue spectral lines correspond to unmatched peaks.

The ability to predict the glycosylation type of a peptide based on its spectral features is beneficial in several ways. First, by breaking the search space into subspaces of N- and O-glycopeptides, the analysis could be performed almost twice as fast, which is a significant improvement in the execution time, especially for large datasets. Moreover, it facilitates data interpretation by distinguishing the glycosylation type when the GPSM includes a glycan composition that belongs to both N- and O- glycan databases. For instance, the glycan portion of the glycopeptide corresponding to the spectrum in Fig. 3 has the composition N3H3F0S0. As reported on the consortium for functional glycomics portal, this composition could potentially represent an N- or O-glycan (Fig. 3). The glycosylation classification model predicts this spectrum to match an O-glycopeptide with high probability (>99%). The peptide portion of this GPSM is in fact a known O-glycosylated peptide (AHEVSEISVRTVYPPEEETGER) that belongs to glycophorin A and has three reported glycosylation sites. Therefore, in this example we know the prediction of the model to be true. This principle holds true for other generic cases as well.

### Application of glycosylation type prediction model to human serum samples

To determine the applicability of the glycosylation classification model in a complex sample, glycopeptides from human serum were enriched using HILIC columns and analyzed by LC-MS/MS. To identify the sample glycopeptides, we conducted two different search approaches and compared the results. First, a conventional approach was used, where N- and O-glycopeptides were searched against the entire oxonium ion containing spectra. In the second approach, the oxonium ion containing spectra were divided between N- and O-linked by the classification model prior to respective searches against the N- and O-glycopeptide databases (Table 1). GPQuest assigned 1065 spectra to 403 unique N-linked glycopeptides that contained 98 peptide backbones and 63 glycan compositions with a glycosylation type classification accuracy of 99.7% using the conventional search (supplementary Table S3). In contrast, 1481 spectra were assigned to N-glycopeptides, where 529 unique N-glycopeptides containing 123 peptide backbones and 80 glycan compositions were identified with a glycosylation type classification accuracy of 100% in the new search approach (supplementary Table S4). Remarkably, the result showed that classifying the spectra to different glycosylation types before conducting the glycoproteomics search increased the number of assigned spectra from 1065 to 1481, a 39% increase. Correspondingly, the number of identified N-glycopeptides increased from 403 to 529, a 31% increase. We then determined the effectiveness of the classification model in identification of O-glycopeptides. It was observed that 200 spectra were assigned to 32 unique O-glycopeptides containing 4 peptide backbones and 31 glycan compositions with a glycosylation type classification accuracy of 96% in the conventional search (supplementary Table S5). However, we did not see an improvement for identification of O-glycopeptides after the glycosylation type classification, where 193 spectra were assigned to 32 unique O- glycopeptides containing 4 peptide backbones and 31 glycan compositions (supplementary Table S6). In summary, the results showed that that pre-classifying the MS/MS spectra into different glycosylation types significantly improved the assignment of N-glycopeptides but appeared to have no effect on O-glycopeptides.

## Conclusion

A central focus of the glycoproteomics field is development of algorithms and software tools for data mining and improved interpretation of large mass spectrometry datasets. To this aim, we have studied the attributes of oxonium ions in the HCD MS/MS spectra of glycopeptides from glycoprotein standards fetuin, glycophorin A and ovalbumin. The pattern of glycan fragment ions depends on not only the composition of glycans, but also the structure of glycan branches. The oxonium ions, resulting from fragmentation of glycans, were shown to be effective in distinguishing MS/MS spectra of N- and O-glycopeptides using machine learning and regression analysis. In glycoprotein standards and human serum samples, the classification achieved an overall accuracy exceeding 90% and facilitated unambiguous identification of about 550 unique glycopeptides at 1% FDR in human serum. This analytical capability has been fully incorporated into the GPQuest software to enable informative data mining. GPQuest is available for downloading at http://www.biomarkercenter.org/GPQuest.

## Additional Information

**How to cite this article**: Eshghi, S. T. *et al*. Classification of Tandem Mass Spectra for Identification of N- and O-linked Glycopeptides. *Sci. Rep.* **6**, 37189; doi: 10.1038/srep37189 (2016).

**Publisher’s note**: Springer Nature remains neutral with regard to jurisdictional claims in published maps and institutional affiliations.

## Supplementary Material

Table S1

Table S2

Table S3

Table S4

Table S5

Table S6

## Figures and Tables

**Table 1:** Glycopeptides identified using two different search approaches. Conventional: all oxonium ions spectra were searched for glycopeptides.

		Assigned MS/MS spectra	Unique glycopeptides	Peptide backbones	Glycan compositions	Accuracy of classification
N-glycopeptides	Conventional	1065	403	98	63	99.7%
	Pre-classification	1481	529	123	80	100%
O-glycopeptides	Conventional	200	32	4	31	96%
	Pre-classification	193	32	4	31	100%

Pre-classification: oxonium ions spectra were separated by classification model and searched for respective N- and O-glycopeptides. The results show that pre-classification of the oxonium ion containing spectra improves the identification of N-glycopeptides, however it does not impact the identification of O-glycopeptides.
